# Fractional lower order linear chirplet transform and its application to bearing fault analysis

**DOI:** 10.1371/journal.pone.0276489

**Published:** 2022-10-21

**Authors:** Junbo Long, Haibin Wang, Hongshe Fan, Zefeng Lao

**Affiliations:** 1 College of Electronic and Engineering, Jiujiang University, Jiujiang, Jiangxi, China; 2 College of Computer and Big Data, Jiujiang University, Jiujiang, Jiangxi, China; Hefei University of Technology, CHINA

## Abstract

The amplitude and frequency of the mechanical bearing fault vibration signals vary with time, and which are non-stationary and non-Gaussian process. The fault signals belong to *α* stable distribution, and the characteristic index 1 < *α* < 2, even the noises are *α* stable distribution in extreme cases. The existing linear chirplet transform (LCT) degenerates, even fails under *α* stable distribution environment. A fractional low order linear chirplet transform (FLOLCT) which takes advantage of fractional *p* order moment is presented for *α* stable distribution noise environment, and the corresponding FLOLCT time-frequency representation (FLOLCTTFR) is developed in this paper. By employing a series of polynomial chirp rate parameters instead of a single chirp rate of the FLOLCT method, a fractional low order polynomial linear chirplet transform (FLOPLCT) is developed to improve time frequency concentration of the signals. The improved FLOLCT and FLOPLCT methods are used to compare with the existing LCT and PLCT methods based on second order statistics, the results reveal performance advantages of the proposed methods. Finally, the FLOLCT and FLOPLCT methods are applied to analyze the fault signature of the bearing ball fault data in the position of DE (Drive end accelerometer) and extract their fault signature, the result illustrates their performances.

## Introduction

Real-time monitoring and fault diagnosis of rotating machinery is an unusual means for normal industrial production. The amplitude and frequency of the mechanical bearing fault vibration signals vary with time, and which are non-stationary. Time-frequency distribution is an effective tool to deal the non-stationary signals, which has been applied for analyzing and processing the bearing fault data [1–3]. A time extraction method was proposed based on S transform to effectively process the pulse signals, which could clearly display the occurrence time of the vibration pulse of rolling bearing fault on the premise of ensuring high time-frequency aggregation [4]. A deformable convolutional neural network time-frequency analysis method was proposed in [5], which could achieve high-precision fault classification for the bearing fault signals. Aimming at the variable speed signals containing multiple faults, Tang et al. proposed a multiple time-frequency curve classification method and classification standard for the fault detection of composite bearings with multiple faults under the condition of time-varying speed without speed resampling [6]. Gültekin et al. used time-frequency images obtained by short-time Fourier transform and multi-perception data fusion based on deep residual network to diagnose the mechanical fault signals, the method combined with different types of measurement signal models can monitor the bearing status more effectively [7].

In time-frequency analysis of the mechanical fault signals, the traditional short time Fourier transform (STFT) time frequency representation method has a low time-frequency resolution [8, 9]. Wavelet Transform (WT) time frequency representation method can take into account both time-domain and frequency-domain resolution, but the limited length of wavelet basis function will cause energy leakage, and it is difficult to obtain accurate time-frequency analysis. Limited by Heisenberg Gabor inequality, the time and frequency of the continuous wavelet transform (CWT) time frequency representation method can not achieve high resolution at the same time, high frequency resolution is necessarily at the expense of time resolution [10, 11]. However, Wigner Ville distribution (WVD) is a bilinear time-frequency analysis method with high time-frequency resolution, but it is easy to lead in cross terms [12]. Steve and Simon first proposed the concept of linear chirplet transform (LCT) based on wavelet transform [13], whereafter, they applied LCT time-frequency representation to a practical problem in Marine radar and achieved good analysis results [14]. The chirp rate of the LCT method is invariable, hence, many experts and scholars have presented many improved post-processing algorithms based on the traditional LCT in recent years [15–23]. Yu Gang et al. proposed a general linear chirplet transform (GLCT) method, but which has a fixed window length, and its resolution is settled [15]. Guan Yunpeng et al. proposed a robust adaptive velocity synchronous linear chirplet transform (VSLCT) time-frequency representation method, which utilized a time-varying window function to improve time-frequency resolution, at the same time, a set of linear frequency modulation wavelets was used to eliminate the smear effect [16]. A polynomial linear chirplet transform (PLCT) time frequency representation method and real-time frequency estimation method using polynomial chirp rate instead of the traditional fixed chirp rate were proposed in [17], which effectively improved the accuracy of frequency estimation. Afterwards, a time-frequency fusion method based on PLCT was proposed in [18], which can characterize the time frequency structure of the signals, nd has better energy concentratioan for the multicomponent signals. The time frequency resolution of PLCT can be improved by synchronous extrusion, and an improved synchrosqueezing polynomial chirplet transform method was proposed [19, 20]. A component matching chirplet transform method based on frequency-dependent chirp rate was proposed in [21], which generates a set of chirplet to match the fault frequency components, so as to realize high resolution time-frequency fault diagnosis. The polynomial estimation of the PLCT method is easily influenced by noise or interference in mechanical fault analysis, so the chirplet transform based on nuclear ridge regression was proposed in [22], which can accurately characterize the time-frequency characteristics of the non-stationary signals and generate the time frequency plane with energy concentration. A group filter-matched signal reconstruction method was proposed using the approximation of source signals with linear chirps at any local time [23].

Due to the repeated transient characteristics caused by local damage, it is easy to be drowned by various interference components and strong noise, so early identification and diagnose of the rolling bearing faults is still difficult. Recently, it is verified that probability density functions (PDFs) of the mechanical bearing fault vibration signals have obviously trailing process, they belong to non-stationary and non-Gaussian distribution *α* stable distribution (1<*α*<2), even the strong noises are also *α* stable distribution [24–26]. The performance of the above-mentioned methods based on second-order statistics degenerates under *α* stable distribution environment, even which fails.

Aiming at the fractional low-order time-frequency distribution methods in *α* stable distribution environment, linear fractional low order short time Fourier transform (FLOSTFT) time-frequency representation method has no cross term interference, but its resolution is not high [27]. The resolution of the linear fractional low order Stockwell transform (FLOST) time-frequency representation method is improved to some extent [28]. Bilinear class fractional low order Wigner Ville distribution (FLOWVD) and fractional low order Wigner Ville distribution (FLOPWVD) time-frequency representation methods have higher time frequency resolution, but there is some cross term interference, the bilinear class fractional low order adaptive kernel time-frequency representation can suppress the cross term interference to some extent, parameter class fractional low order autoregressive (FLOAR), fractional low order moving average (FLOMA) and fractional low order autoregressive moving average (FLOARMA) time-frequency methods have no cross term, but the calculation is large and their resolution is not high [29, 30]. Hence, we propose the improved franctional low order linear chirplet transform (FLOLCT) and franctional low order polynomial linear chirplet transform (FLOPLCT) methods inspired by the LCT and PLCT methods in this paper, the corresponding franctional low order linear chirplet transform time frequency representation (FLOLCTTFR) and franctional low order polynomial linear chirplet transform time frequency representation (FLOPLCTTFR) are introduced. The improved FLOLCT and FLOPLCT methods and the traditional LCT and PLCT methods employing second order statistics are compared under Gaussian and *α* stable distribution environments. The simulation results show that the proposed FLOLCTTFR and FLOPLCTTFR methods have performance advantages and higher time frequency resolution, and can better be suitable for the impulse noise environment than the LCT and PLCT methods. The estimated *MSEs* of the polynomial parameters employing the FLOPLCT time frequency representation method are smaller than the PLCT method under different *α*(*α* < 2) and *MSNR*. Finally, we apply the improved FLOLCTTFR and FLOPLCTTFR methods to analyze the bearing ball fault in the position of DE data contaminated by Gaussian noise and *α* stable distribution, the results indicate that the performance of the improved methods is better than the existing methods, and which are feasible, effective and robust for fault diagnosis.

In this paper, the improved FLOLCTTFR and FLOPLCTTFR technologies based on fractional lower order statistics and LCT are proposed for the bearing ball fault in the position of DE data analysis under Gaussian and *α* stable distribution environment. The paper is structured in the following manner. *α* stable distribution description, bearing fault data and their *α* stable distribution model parameter estimation are introduced in section 2. The improved fractional lower order linear chirplet transform and fractional lower order polynomial linear chirplet transform methods are demonstrated in Section 3, and simulation comparisons with the existing LCT and PLCT methods based on second order statistics are performed to demonstrate superiority of the improved methods. Applications of the improved methods for the bearing ball fault data in the position of DE are demonstrated in section 4. Finally, conclusions and future research are given in Section 5.

## *α* stable distribution and bearing fault data

### *α* stable distribution description

**Fig 1:**
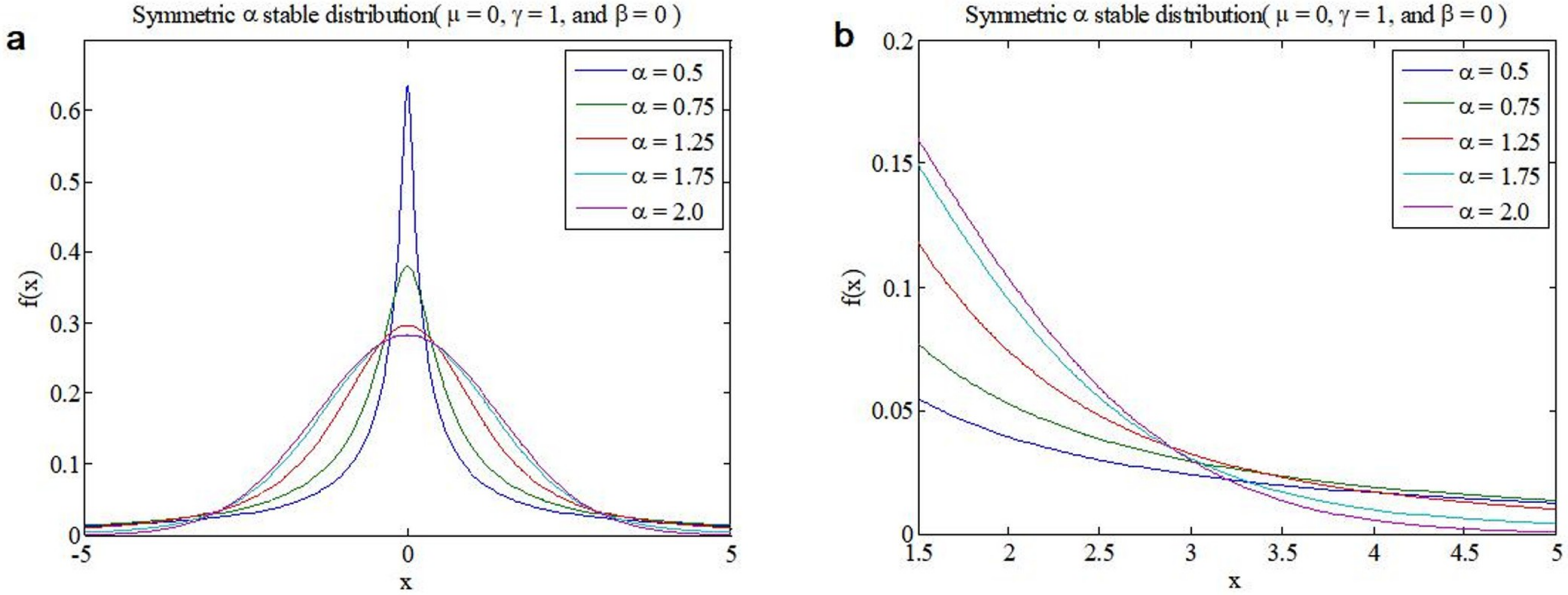
PDFs of *SαS* stable distribution under *α* = 0.5, 0.75, 1.25, 1.75 and 2.0. (a. Full PDFs diagram; b. Local trailing PDFs diagram).

The characteristic function of *α* stable distribution can be written by

$$\varphi (t)=\text{exp}\left\{j\;\mu t-\gamma {\left|t\right|}^{\alpha }[1+j\beta sign(t)\omega (\tau ,\alpha )]\right\}$$ (1)

where *α* is the characteristic index, when *α* = 2, Eq (1) degenerate into Gaussian distribution, hence which is often called the generalized Gaussian distribution. *μ*, *γ* and *β* are the location parameter, the dispersion coefficient and the symmetry parameter, respectively. If *μ* = 0 and *γ* = 1, the α stable distribution is called the standard *α* stable distribution, and if *μ* = 0, *γ* = 1 and *β* = 0, it is called the standard symmetric *α* stable distribution (*SαS*). Probability density functions (PDFs) of *SαS* stable distribution under *α* = 0.5, 1.0, 1.5 and 2.0 are shown in Fig 1. It can be seen that the smaller the characteristic index *α*, the stronger the impulsivity, and their PDFs have a long tail.

### Bearing fault data and their *α* stable distribution parameter estimation

The real bearing fault data are gotten from the Case Western Reserve University (CWRU) bearing data center [31], and the data includes the normal bearings data, drive end bearing fault data and fan end bearing fault data. The fault points include inner race fault, ball fault, and outer race position relative to load zone centered at 6:00, 3:00 and 12:00. The fault diameter is 0.021 inches, the sampling frequency of the data is 12000Hz, and the motor speed is set at 1746–1752 revolutions per minute (rpm). The acceleration data is measured at locations near to base accelerometer (BA), the drive end accelerometer (DE) and fan end accelerometer (FE).

**Fig 2:**
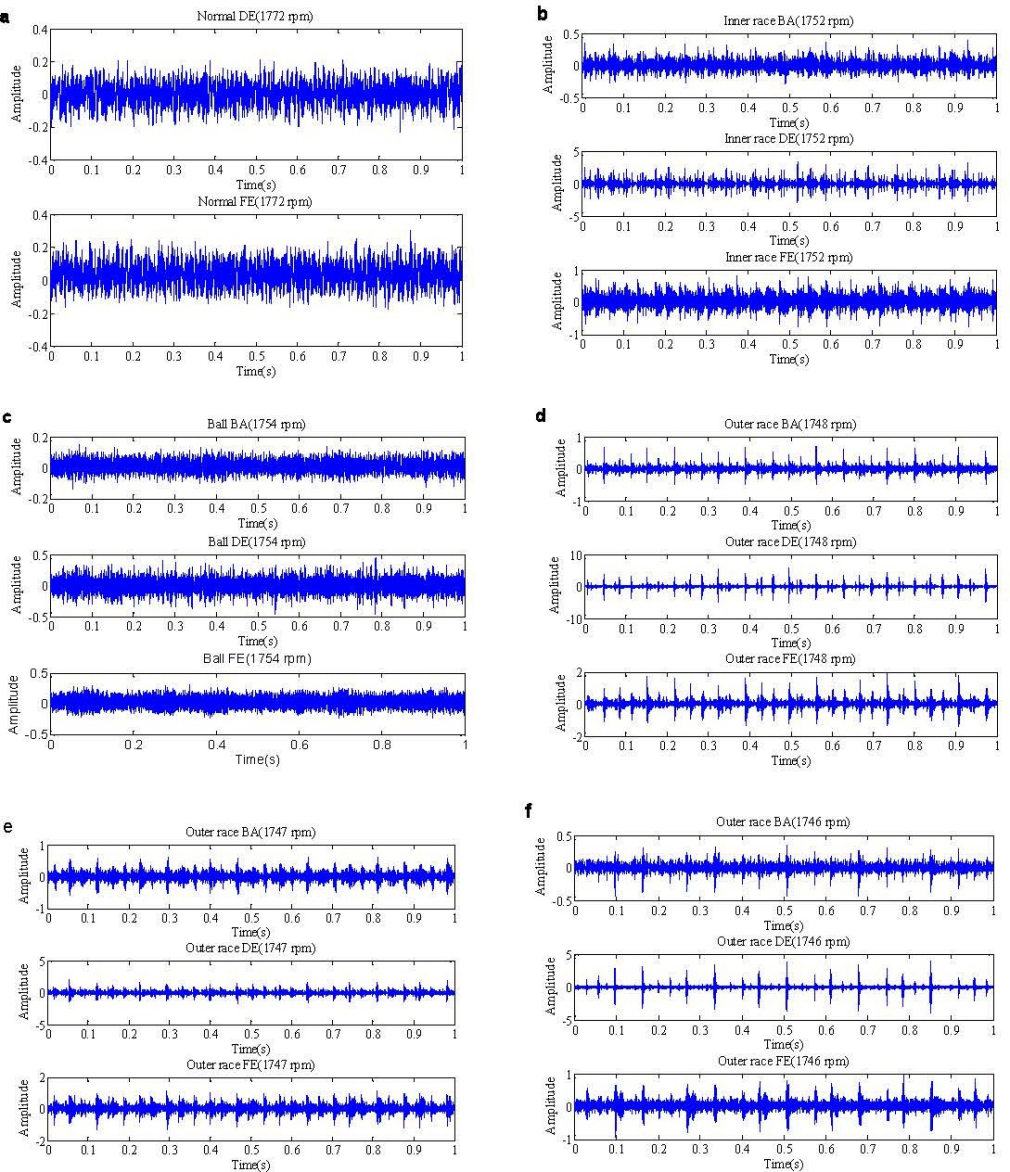
The waveforms of the normal and drive end bearing fault data. (a. The waveforms of the normal DE and FE data; b. The waveforms of the inner race BA, DE and FE fault data; c. The waveforms of the ball BA, DE and FE fault data; d. The waveforms of the outer race BA, DE and FE fault data position relative to load zone centered at 6:00; e. The waveforms of the outer race BA, DE and FE fault data position relative to load zone orthogonal at 3:00; f. The waveforms of the outer race BA, DE and FE fault data position relative to load zone opposite at 12:00.

The normal and drive end bearing fault data are selected as the test signals in this paper. The waveforms of the normal and drive end bearing fault data are shown in Fig 2. We apply *α* stable distribution statistical model in Eq (1) to estimate the *α* stable distribution parameters of the normal signals and the drive end bearing fault signals including the inner race fault, ball fault and outer race fault data, the results are given in Table 1. The result shows that the parameters *α* of the normal BE and FE data and the ball BA and FE fault data *α* = 2, and they belong to Gaussian distribution. However, the parameters *α* of the inner race fault and outer race position relative to load zone centered at 6:00, orthogonal at 3:00 and opposite at 12:00 *α* < 2, they are *α* stable distribution.

**Table 1:** *α* stable distribution parameter estimation of the normal and drive end bearing fault data.

Bearing Fault		accelerometer data	*α* stable parameters			
			*α*	*Β*	*γ*	*μ*
Normal		DE	2	-1	0.0452	0.0136
		FE	2	0.9365	0.0485	0.031
Inner Race		BA	1.6843	0.1813	0.0434	0.0051
		DE	1.3731	0.046	0.2123	0.007
		FE	1.7196	0.0872	0.1056	0.034
Ball		BA	2	0.5306	0.0259	0.0092
		DE	1.9479	-0.1072	0.0722	0.0041
		FE	2	0.0525	0.0546	0.0294
Outer Race Position Relative to Load Zone	Centered @6:00	BA	1.5611	-0.1084	0.0433	0.0035
		DE	1.092	-0.0069	0.1214	-0.0022
		FE	1.4042	-0.0089	0.0822	0.0354
	Orthogonal @3:00	BA	1.6398	0.0067	0.0768	0.007
		DE	1.5505	0.0341	0.1441	0.0038
		FE	1.5333	0.0414	0.1181	0.035
	Opposite @12:00	BA	1.7086	0.1632	0.0334	0.0069
		DE	1.3755	0.0555	0.0782	0.0076
		FE	1.6325	-0.0591	0.0689	0.0069

**Fig 3:**
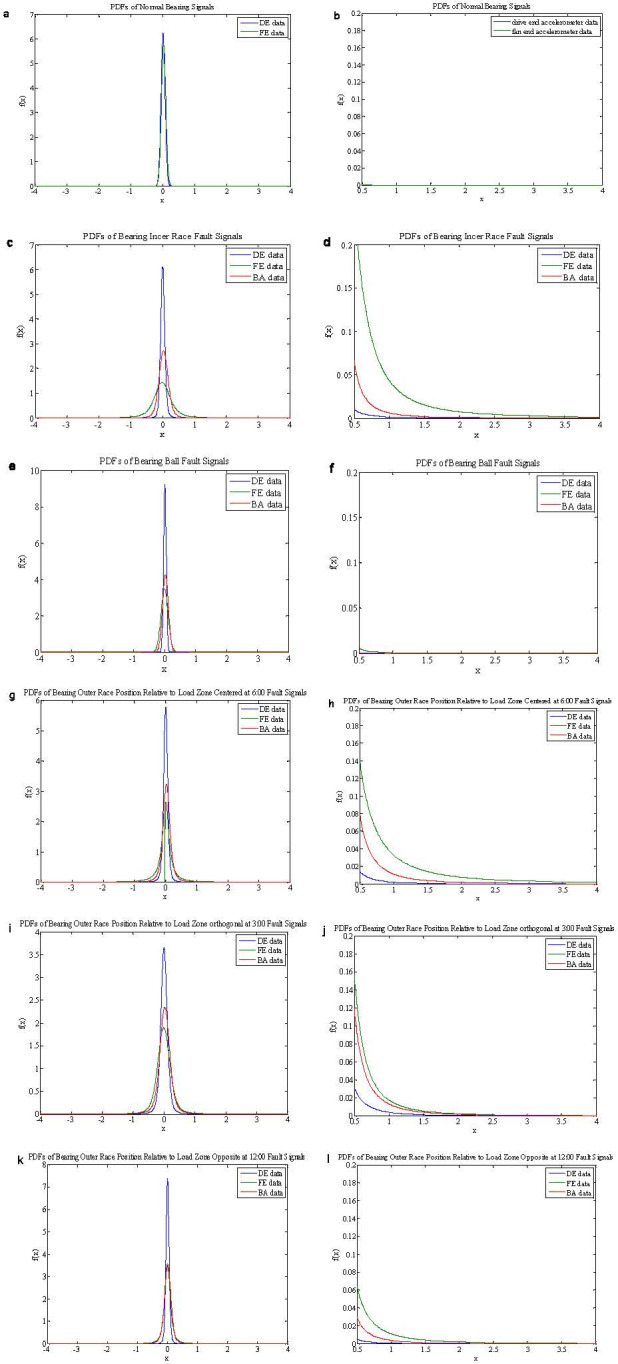
PDFs of the normal data and drive end bearing fault data. (a-b. PDFs of the normal data; c-d. PDFs of the inner race BA, DE and FE fault data; E-F. PDFs of the ball BA, DE and FE fault data; g-h. PDFs of the outer race BA, DE and FE fault data position relative to load zone centered at 6:00; i-j. PDFs of the outer race BA, DE and FE fault data position relative to load zone orthogonal at 3:00; k-l. PDFs of the outer race BA, DE and FE fault data position relative to load zone opposite at 12:00.).

PDFs of the normal and drive end bearing fault data in Table 1 are shown in Fig 3. It is seen that PDFs of the inner race fault and outer race data have long trails, and their pulse characteristics are quite remarkable. But PDFs of the normal data and ball fault data have no tails and pulse characteristics.

## Fractional lower order linear chirplet transform methods

### FLOLCT time-frequency representation method

#### Principle

We have proposed fractional lower order short time Fourier transform (FLOSTFT) of a signal *x*(*τ*) in [29]

$$FLOSTFT(t,\omega )=\int_{-\infty }^{+\infty }x^{<P>}(\tau )h(\tau -t)e^{-j\omega \tau }d\tau$$ (2)

where < *p* > denotes *p* order moment of *x*(*τ*), *p* is a real coefficient and *α* < *p* ≤ 2, *α* is characteristic index of *α* stable distribution noise, and $x^{<p>}(\tau )={\left|x(\tau )\right|}^{p-1}\cdot sign[x(\tau )]$, $sign\left[x(\tau )\right]=\left\{\begin{array}{c} 1\begin{array}{cc} & x(\tau )>0 \end{array}\\ 0\begin{array}{cc} & x(\tau )=0 \end{array}\\ -1\begin{array}{cc} & x(\tau )<0 \end{array} \end{array}\right.$. The corresponding fractional lower order short time Fourier transform time-frequency representation (FLOSTFTTFR) of the signal *x*(*τ*) can be written by

$$FLOSTFTTFR(t,\omega )={\left|FLOSTFT(t,\omega )\right|}^{2}={\left|\int_{-\infty }^{+\infty }x^{<P>}(\tau )h(\tau -t)e^{-j\omega \tau }d\tau \right|}^{2}$$ (3)

According to the definition of the FLOSTFT in Eq (2) and liner chirplet transform (LCT) [13, 14], we define fractional lower order liner chirplet transform (FLOLCT) as

$$FLOLCT(t,\omega ,c,\sigma )=\int_{-\infty }^{+\infty }x^{<p>}(\tau )h_{\sigma }(\tau -t)\Psi_{\left(t,c\right)}(\tau )e^{-j\omega \tau }d\tau =\int_{-\infty }^{+\infty }\bar{x}\Psi_{\left(t,c\right)}(\tau )e^{-j\omega \tau }d\tau$$ (4)

$$\bar{x}=x^{<p>}(\tau )h_{\sigma }(\tau -t)$$ (5)

$$\Psi_{\left(t,c\right)}(\tau )=e^{-\frac{1}{2}jc{\left(\tau -t\right)}^{2}}$$ (6)

where, the parameters *c* and *t* denote the chirp rate and time, respectively. **$\bar{x}$** stands for a windowed fractional lower *p* order moment function, and *h*_*σ*_(*τ*—*t*) is a real window function. Ψ_(*t*, *c*)_(*τ*) is the discrete demodulated function. Fractional low order linear chirplet transform is the extension of fractional low order short time Fourier transform, and it has the same form of Fourier transform calculation as the fractional low order short time Fourier transform. FLOLCT is usually obtained by Gaussian frequency FM wavelet packet, so it is often called fractional low order Gaussian linear chirplet transform.

Eq (6) can be written by

$$\Psi_{\left(t,c\right)}(\tau )=e^{-\frac{1}{2}jc{\left(\tau -t\right)}^{2}}=e^{jct\tau -\frac{1}{2}jc\tau^{2}-\frac{1}{2}jct^{2}}=e^{jct\tau }e^{-\frac{1}{2}jc\tau^{2}}e^{-\frac{1}{2}jct^{2}}$$ (7)

where *e*^*jctτ*^ is frequency shift operating factor, and its amplitude is *ct*. $e^{-\frac{1}{2}jc\tau^{2}}$ is frequency rotation operation factor, and its rotation angle is *θ* = arctan(-*c*).

The corresponding fractional lower order linear chirplet transform time frequency representation (FLOLCTTFR) can be written by

$$\begin{array}{l} FLOLCTTFR(t,\omega ,c,\sigma )={\left|FLOLCT_{x}^{}(t,\omega ,c,\sigma )\right|}^{2}={\left|\int_{-\infty }^{+\infty }\bar{x}\Psi_{\left(t,c\right)}(\tau )e^{-j\omega \tau }d\tau \right|}^{2}\\ ={\left|\int_{-\infty }^{+\infty }x^{<p>}(\tau )\frac{1}{\sqrt{2\pi }\sigma }e^{-\frac{1}{2}{\left(\frac{\tau -t}{\sigma }\right)}^{2}}e^{jct\tau }e^{-\frac{1}{2}jc\tau^{2}}e^{-\frac{1}{2}jct^{2}}e^{-j\omega \tau }d\tau \right|}^{2}\\ ={\left|\int_{-\infty }^{+\infty }x^{<p>}(\tau )\frac{1}{\sqrt{2\pi }\sigma }e^{-\frac{1}{2}{\left(\frac{\tau -t}{\sigma }\right)}^{2}}e^{jct\tau }e^{-\frac{1}{2}jc\tau^{2}}e^{-j\omega \tau }d\tau \right|}^{2}\\ ={\left|\int_{-\infty }^{+\infty }\bar{x}\tilde{\Psi }(t,c)e^{-j\omega \tau }d\tau \right|}^{2} \end{array}$$ (8)

$$\tilde{\Psi }(t,c)=e^{jct\tau }e^{-\frac{1}{2}jc\tau^{2}}$$ (9)

The value of the chirp rate *c* directly affects energy concentration of the analytical signal *x*(*τ*) in time-frequency domain. The closer *c* is to the actual frequency of the analytical signal, the higher the energy concentration is in time-frequency domain. When the chirp rate *c* = 0, then ψ_(*t*, *c*)_(*τ*) = 1 and $\tilde{\Psi }(t,c)=1$, Eq (4) changes into Eq (3), and the FLOLCT time-frequency method degrades to FLOSTFT time-frequency method. When *p* = 2, FLOLCT in Eq (4) degenerates into LCT, and FLOCTTFR in Eq (8) changes into LCTTFR. Hence, the LCT method is a special case of the FLOLCT method, and FLOLCT is a generalized LCT.

#### Application review

In this simulation, the mixed signal contaminated by *v*(*t*)(Gaussian or *SαS* stable distribution noise) is defined as

$$y(\tau )=\sin\left[2\pi (\frac{1}{120}t^{4}+\frac{1}{9}t^{3}+2t^{2}+30t)\right]+v(t)=x(\tau )+v(t)$$ (10)

If *v*(*t*) is Gaussian noise, we apply signal to Gaussian noise ratio (*SNR*), but if *v*(*t*) is *α* stable distribution noise, there is no finite second moment, the variance of *α* stable distribution noise becomes meaningless, and *SNR* is unusable. So mixed signal noise ratio (*MSNR*) or generalized signal noise ratio (*GSNR*) can be used to replace *SNR*, and *MSNR* and *GSNR* be written by $MSNR=10\log_{10}(\sigma_{x}{}^{2}/\gamma )$ and $GSNR=10\log_{10}\left\{\sum_{n=1}^{N}{\left|x(n)\right|}^{2}/N\gamma \right\}$, respectively. Where *σ*_*x*_ is variance of the signal *x*(*τ*), *N* is sample size, and *γ* is dispersion coefficient of the *α* stable distribution noise. In this paper, we use *MSNR* to perform the following experiments.

**Fig 4:**
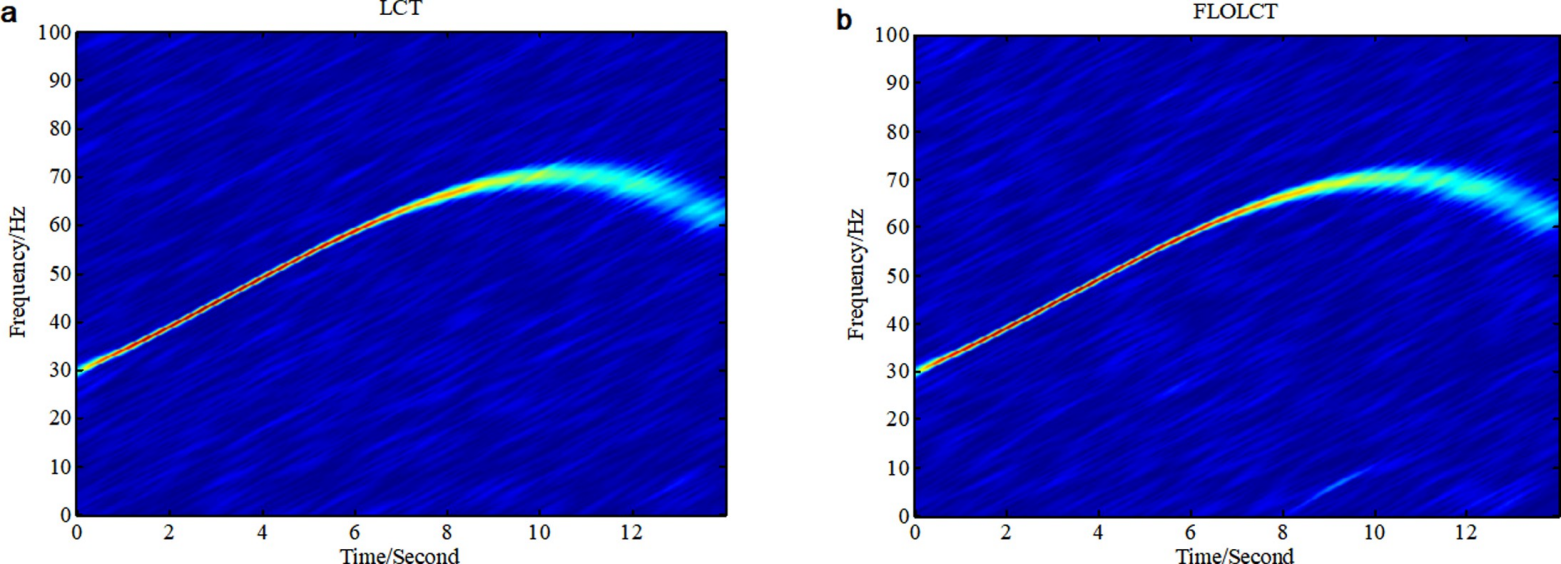
Time-frequency representations of the signal *x*(*τ*) in Gaussian noise environment (*c* = 6, *SNR* = 6*dB*). (a. LCT time-frequency representation of the signal *x*(*τ*); b. FLOLCT time-frequency representation of the signal *x*(*τ*)).

**Fig 5:**
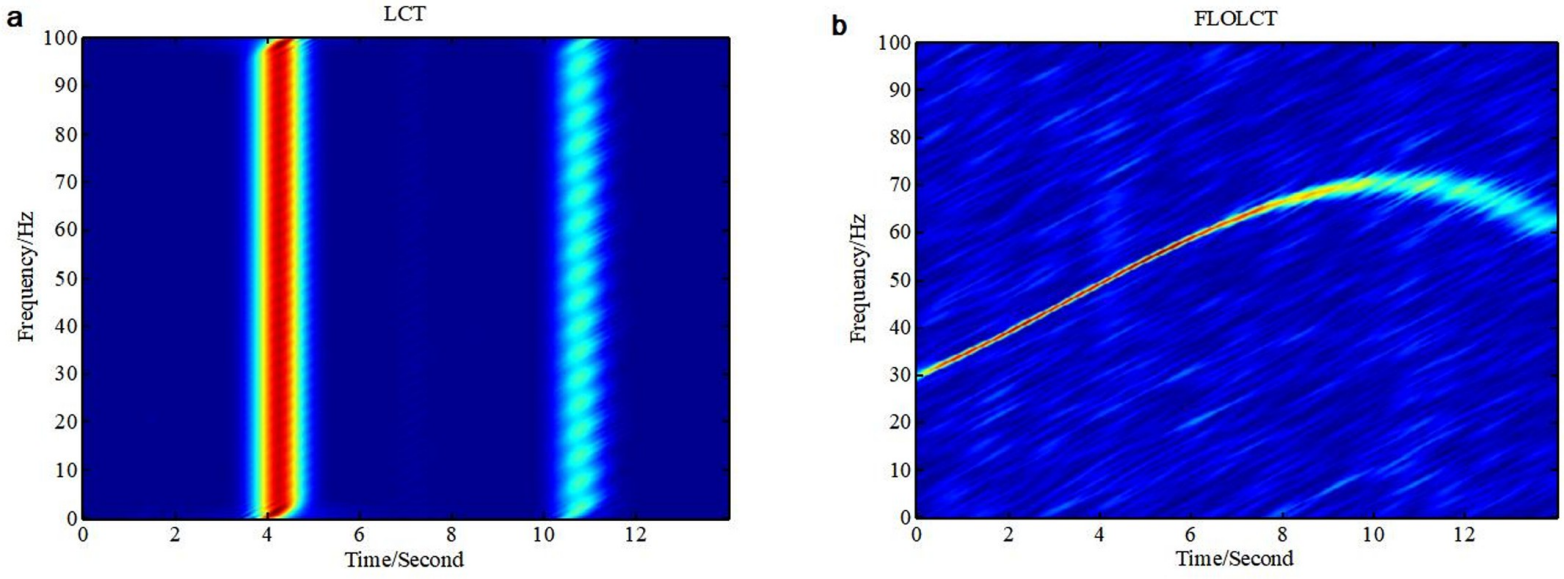
Time-frequency representations of the signal *x*(*τ*) in *α* stable distribution noise environment (*MSNR* = 16*dB*, *α* = 0.8). (a. LCT time frequency representation of the signal *x*(*τ*); b. FLOLCT time-frequency representation of the signal *x*(*τ*)).

We apply the existing LCT (*c* = 6) method and the improved FLOLCT (*c* = 6, *p* = 1.2) method to estimate the time-frequency representations of the signal *x*(*t*)(Eq 10) in Gaussian noise environment (*SNR* = 6*dB*) and *α* stable distribution noise environment (*MSNR* = 16*dB*, *α* = 0.8), the simulation results are shown in Figs 4 and 5.

#### Remarks

Fig 4A and 4B are the time frequency representations of the signal *x*(*τ*) employing LCT (*c* = 6) and FLOLCT (*c* = 6, *p* = 1.2) in Gaussian noise environment (*SNR* = 6*dB*), respectively. The LCT and FLOLCT time frequency distributions of the signal *x*(*τ*) are shown in Fig 5A and 5B in *α* stable distribution noise environment (*α* = 0.8, *MSNR* = 16*dB* and *p* = 1.2), respectively. It can be seen that the LCT method can only work in Gaussian noise environment, but the FLOLCT method can work in Gaussian noise environment and *α* stable distribution noise environment, and which has good toughness.

The FLOLCT can provide a well-concentrated time frequency representation for the liner modulated signals, the FLOLCT method has better resolution for the multi frequency modulation signals than the FLOSTFT method because of the use of chirp rate *c*. However, the time frequency resolution of the FLOLCT method is controlled by the chirp rate *c*, the closer the chirp rate *c* is to the true instantaneous frequency of the signal, the higher energy concentration of the signal in time-frequency domain. Aimming at the signals containing time varying frequency component, their energy concentration are not satisfactory because that the FLOLCT method has immobile chirp rate *c*, as shown in Figs 4B and 5B. In the part of time 0–8 seconds, the frequency resolution of the signal *x*(*τ*) is very high, but the signal *x*(*τ*) has very low time-frequency resolution in the part of time 8–14 seconds.

### FLOPLCT time-frequency representation method

#### Principle

FLOLCT in section 3.1 has invariable chirp rate *c*. When the instantaneous frequency trajectory of the signal is a linear function of time, which has high energy aggregation in time frequency plane. But when the instantaneous frequency trajectory of the signal is not a linear function of time, FLOLCT will not adapt to the frequency shift of the signal. Hence, a series of polynomial chirp rate parameters *c*_1_, *c*_2_ … *c*_*n*-1_, *c*_*n*_ can be used to replace the invariable chirp rate *c*.

Fractional lower order polynomial linear chirplet transform (FLOPLCT) of a signal *x*(*τ*) can be written as

$$\begin{array}{l} FLOPLCT(t,\omega ,\tilde{c},\sigma )=\int_{-\infty }^{+\infty }x^{<p>}(\tau )h_{\sigma }(\tau -t)\Psi (\tau ,t,\tilde{c})e^{-j\omega \tau }d\tau \\ =\int_{-\infty }^{+\infty }\bar{x}\Psi (\tau ,t,\tilde{c})e^{-j\omega \tau }d\tau \end{array}$$ (11)

$$\begin{array}{l} \Psi (\tau ,t,\tilde{c})=e^{j\sum_{m=2}^{n+1}(\tau c_{m-1}t^{m-1}-\frac{1}{m}c_{m-1}\tau^{m})}\\ =e^{j\sum_{m=2}^{n+1}\tau c_{m-1}t^{m-1}}e^{-j\sum_{m=2}^{n+1}\frac{1}{m}c_{m-1}\tau^{m}} \end{array}$$ (12)

where $\tilde{c}=c_{1},c_{2}\ldots c_{n-1},c_{n}$, $\bar{x}=x^{<p>}(\tau )h_{\sigma }(\tau -t)$. $e^{j\sum_{m=2}^{n+1}\tau c_{m-1}t^{m-1}}$ is nonlinear frequency shift operating factor, and its frequency increment amplitude is $\sum_{m=2}^{n+1}c_{m-1}t^{m-1}$. $e^{-j\sum_{m=2}^{n+1}\frac{1}{m}c_{m-1}\tau^{m}}$ is nonlinear frequency rotation operation factor, the magnitude of the signal’s first rotation is determined by the signal’s own immediate frequency. When *n* = 1, the polynomial chirp rate parameters $\tilde{c}=c_{1}$, then FLOPLCT degenerates into FLOLCT. The corresponding fractional lower order polynomial liner chirplet transform time frequency representation (FLOPLCTTFR) can be written by

$$FLOPLCTTFR(t,\omega ,\tilde{c},\sigma )={\left|FLOPLCT(t,\omega ,\tilde{c},\sigma )\right|}^{2}={\left|\int_{-\infty }^{+\infty }\bar{x}\Psi (\tau ,t,\tilde{c})e^{-j\omega \tau }d\tau \right|}^{2}$$ (13)

Next, we will talk about how to solve the polynomial chirp rate parameters $\tilde{c}=c_{1},c_{2}\ldots c_{n-1},c_{n}$. Firstly, let $\tilde{c}=0,0\ldots 0,0$, and substitute it into Eq (13) to compute fractional lower order polynomial liner chirplet transform time frequency representation *FLOPLCTTFR*_1_ of the signal *x*(*τ*), Locate higher energy concentration points in *FLOPLCTTFR* time frequency plane. Approximate the higher energy concentration points with a *n* order polynomial function, then the polynomial chirp rate parameters estimation $\bar{\tilde{c}}={\bar{c}}_{1},{\bar{c}}_{2}$ can be gotten. Substitute $\bar{\tilde{c}}={\bar{c}}_{1},{\bar{c}}_{2}$ into Eq (13), get *FLOPLCTTFR*_2_, and calculate |*FLOPLCTTFR*_2_—*FLOPLCTTFR*_1_|, we can set a threshold coefficient *ξ*, if

$$\left|FLOPLCTTFR_{n}-FLOPLCTTFR_{n-1}\right|<\xi$$ (14)

The gotten parameters $\bar{\tilde{c}}={\bar{c}}_{1},{\bar{c}}_{2}\ldots {\bar{c}}_{n-1},{\bar{c}}_{n}$ will be used as the final estimation coefficients. Finally, $\bar{\tilde{c}}={\bar{c}}_{1},{\bar{c}}_{2}\ldots {\bar{c}}_{n-1},{\bar{c}}_{n}$ are substituted into Eq (13), FLOPLCTTFR of the signal *x*(*τ*) is gotten.

#### Application review

In this simulation, the mixed signal *x*(*τ*) contaminated by *v*(*t*)(Gaussian or *SαS* stable distribution noise) in Eq (10) are used as the test signal. It can be seen that the order *n* = 4 of the polynomial signal *x*(*τ*) in Eq (10), and $\tilde{c}=c_{1},c_{2},c_{3},c_{4}=[30,4,1/3,1/30]$. The mixed mean square error (*MSE*) is defined in Eq (15), where, *K* is the number of Monte-Carlo experiment, *c*_*n*_ is the real parameters, and ${\bar{c}}_{n}$ is the estimated parameters employing the PLCT or FLOPLCT method.


$$MSE=10\log_{10}\left\{\frac{1}{K}\sum_{k=1}^{K}\sum_{n=1}^{4}{\left[c_{n}-{\bar{c}}_{n}\right]}^{2}\right\}$$ (15)

**Fig 6:**
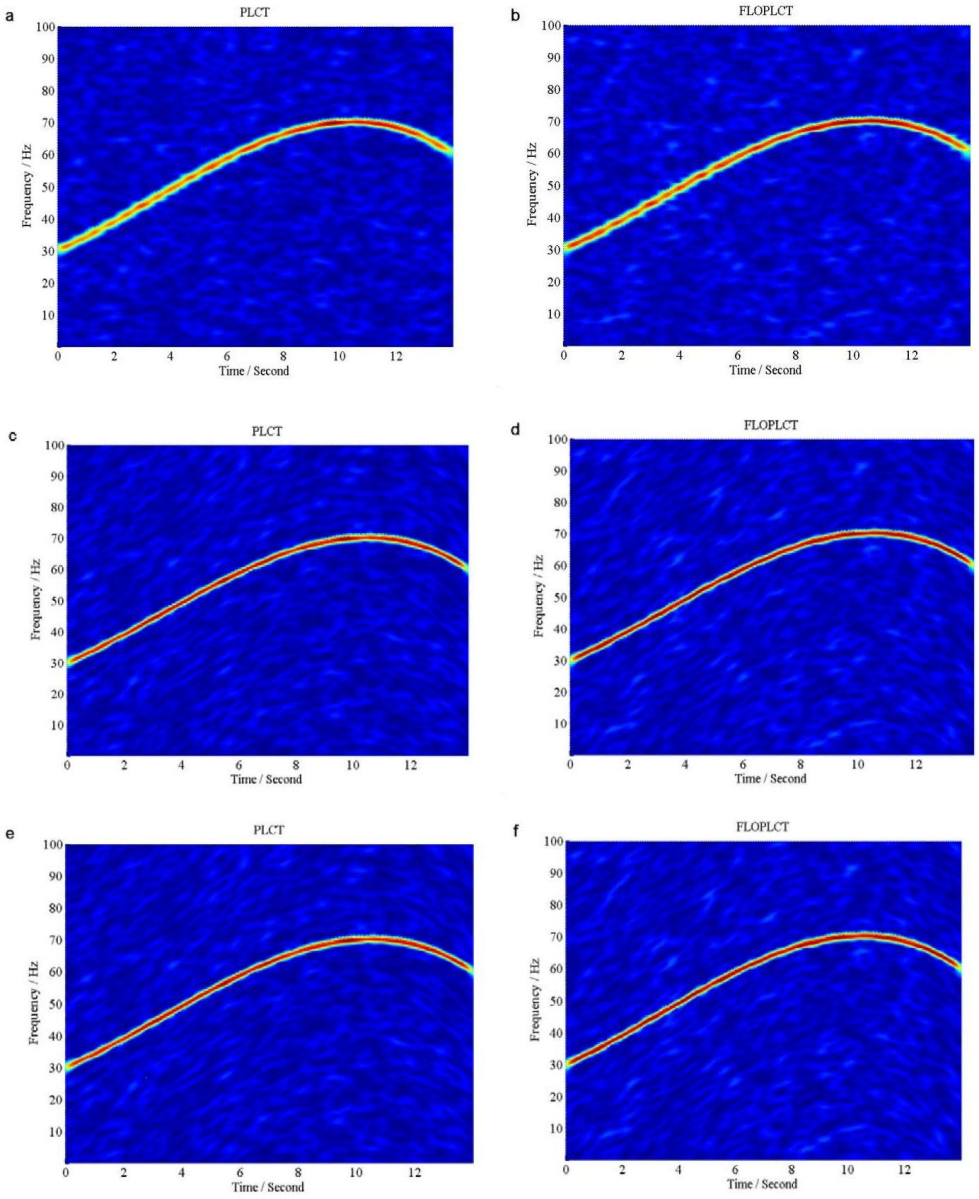
Time-frequency representations of the signal *x*(*τ*) in Gaussian noise environment (*SNR* = 5*dB*). (a. The first time PLCT time-frequency representation of the signal *x*(*τ*); b. The first time FLOPLCT time-frequency representation of the signal *x*(*τ*); c. The second time PLCT time-frequency representation of the signal *x*(*τ*); d. The second time FLOPLCT time-frequency representation of the signal *x*(*τ*); e. The third time PLCT time-frequency representation of the signal *x*(*τ*); f. The third time FLOPLCT time-frequency representation of the signal *x*(*τ*)).

**Fig 7:**
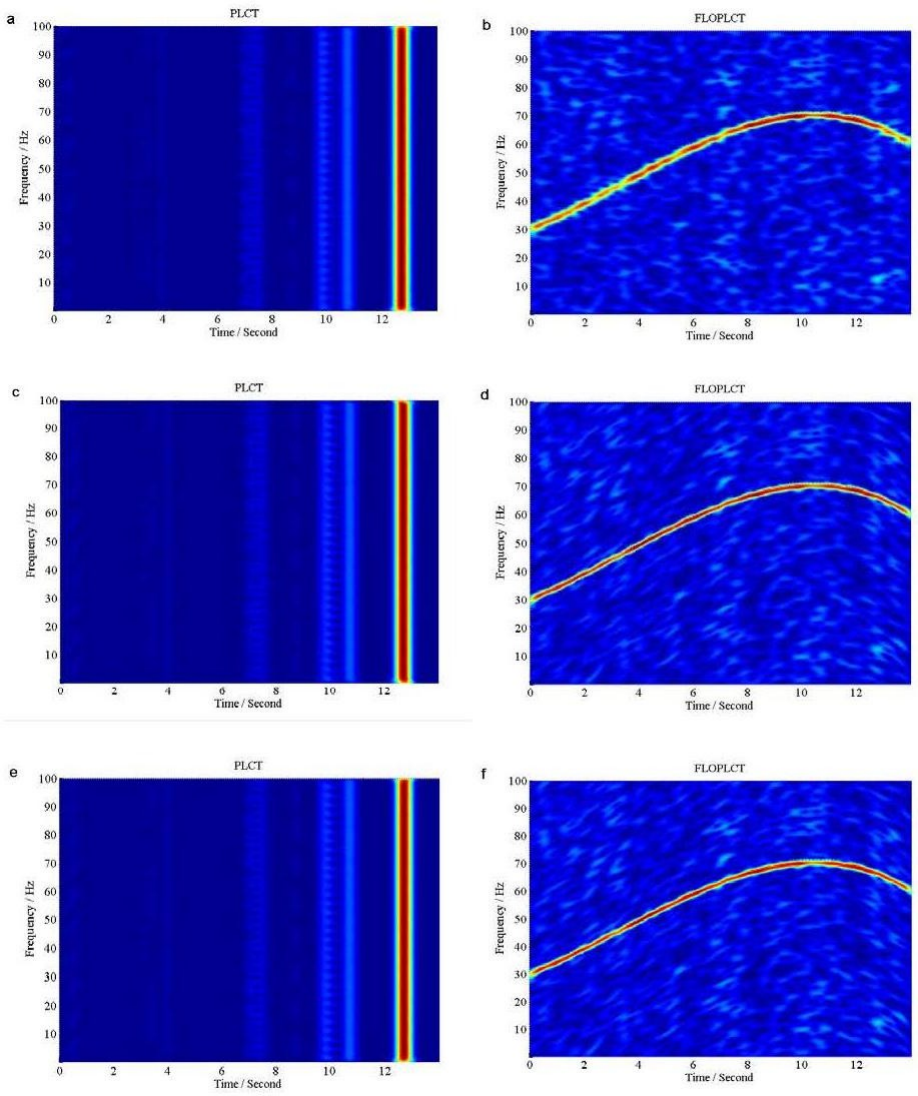
Time-frequency representations of the signal *x*(*τ*) in *α* stable distribution noise environment (*MSNR* = 16*dB*, *α* = 0.8). (a. The first time PLCT time-frequency representation of the signal *x*(*τ*); b. The first time FLOPLCT time-frequency representation of the signal *x*(*τ*); c. The second time PLCT time-frequency representation of the signal *x*(*τ*); d. The second time FLOPLCT time-frequency representation of the signal *x*(*τ*); e. The third time PLCT time-frequency representation of the signal *x*(*τ*); f. The third time FLOPLCT time-frequency representation of the signal *x*(*τ*)).

If *v*(*t*) is Gaussian noise, we use signal to Gaussian noise ratio (*SNR*), but if *v*(*t*) is *α* stable distribution noise, we use *MSNR* to perform the following experiments. The initial value of the polynomial chirp rate parameters is [0 0 0 0], three times PLCT and FLOPLCT (*n* = 4, *p* = 1.2) time-frequency representation of the signal *x*(*τ*) are shown in Fig 6A–6F under Gaussian noise environment (*SNR* = 5*dB*), respectively, and the estimated three times polynomial chirp rate parameters of the PLCT and FLOPLCT time-frequency distribution methods are given Table 2. Fig 7A–7F are three times (*n* = 4) time-frequency representations of the signal *x*(*τ*) employing the PLCT and FLOPLCT (*p* = 1.2) methods in *α* stable distribution noise environment (*MSNR* = 16*dB*, *α* = 0.8), and Table 3 is the parameters estimation of three times polynomial chirp rate.

**Table 2:** The estimated polynomial parameters of the PLCT and FLOPLCT methods in Gaussian noise environment (*SNR* = 5*dB*).

Polynomial parameters		PLCT			FLOPLCT		
Real *c*	Estimated *c*	n = 1	n = 2	n = 3	n = 1	n = 2	n = 3
30.00000	*c*_*1*_	30.08907	30.01061	30.01075	30.08394	30.00318	30.00443
4.00000	*c*_*2*_	3.98387	3.99532	3.99404	3.97367	3.99755	3.99597
0.33333	*c*_*3*_	0.33051	0.33404	0.33439	0.33268	0.33384	0.33423
0.03333	*c*_*4*_	-0.03301	-0.03337	-0.03339	-0.03311	-0.03336	-0.03338

**Table 3:** The estimated polynomial parameters of the PLCT and FLOPLCT methods in *α* stable distribution noise environment (*MSNR* = 16*dB*, *α* = 0.8).

Polynomial parameters		PLCT			FLOPLCT		
Real c	Estimated c	n = 1	n = 2	n = 3	n = 1	n = 2	n = 3
30.00000	*c*_*1*_	33.34755	30.44407	29.38530	30.06923	29.99945	30.00018
4.00000	*c*_*2*_	7.78413	4.66236	5.80617	4.00091	3.99742	3.99665
0.33333	*c*_*3*_	-0.54496	0.15859	-0.09421	0.32692	0.33395	0.33412
0.03333	*c*_*4*_	0.01095	-0.02324	-0.00869	-0.03281	-0.03336	-0.03337

**Fig 8:**
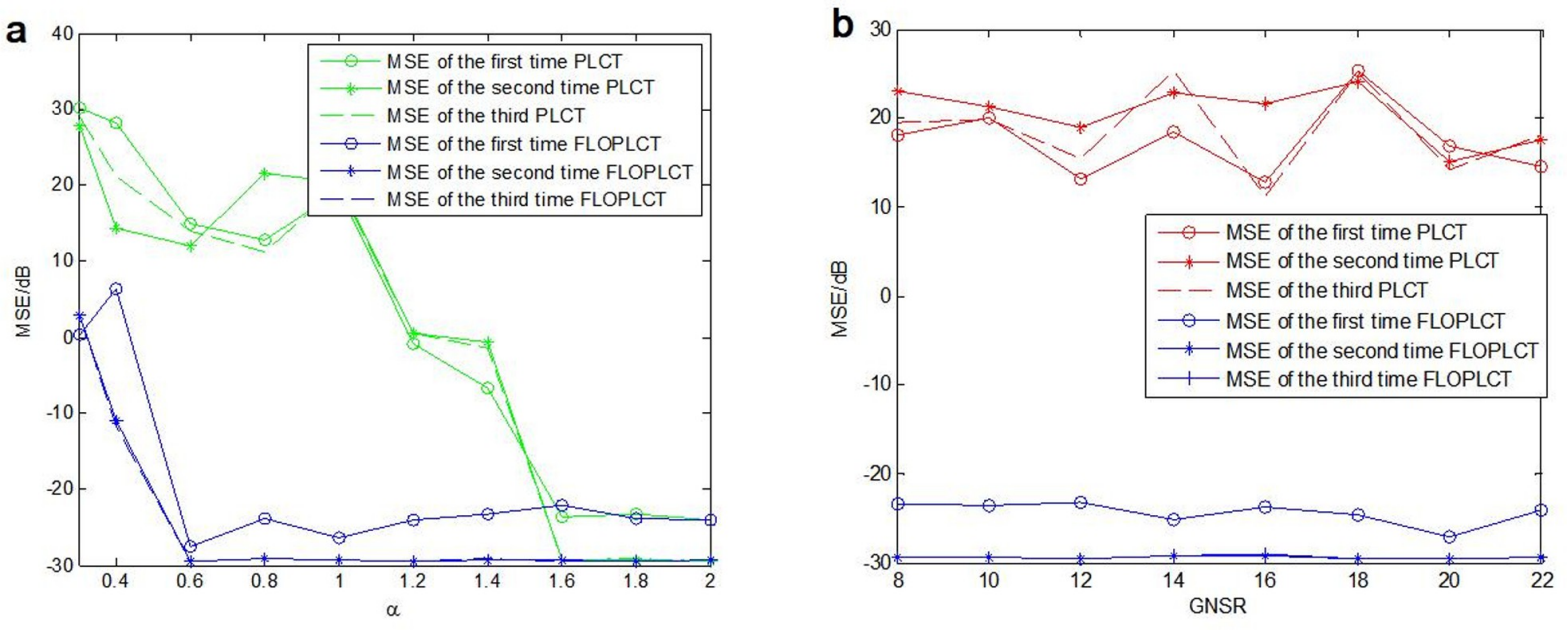
The mixed *MSEs* comparisons of the polynomial chirp rate parameters of the PLCT and FLOPLCT methods in different *α* and *MSNR*. (a) *MSNR* = 16*dB*, *n* = 4, *p* = 1.2, *K* = 20, *α* changes from 0.3 to 2; (b) *α* = 0.8, *n* = 4, *p* = 1.2, *K* = 20, *GSNR* changes from 8*dB* to 22*dB*.

To further verify the advantages of the proposed FLOPLCT method, the detailed comparison experiments with the PLCT method are done under *α* stable distribution noise environment using different characteristic index *α* and *MSNR*, respectively. We use *y*(*τ*) in Eq (13) as the experimental signal, let the characteristic index *α* changing form 0.3 to 2 (when *α* = 2, *v*(*τ*) is Gaussian noise), *MSNR* = 16*dB*, *n* = 4, *p* = 1.2, *K* = 20, *MSEs* of three times polynomial parameters estimation employing the PLCT and FLOPLCT methods are shown in Fig 8A, respectively. Let *α* = 0.8, *n* = 4, *p* = 1.2, *K* = 20, *MSNR* changes from 8*dB* to 22*dB*, *MSEs* of three times polynomial parameters estimation employing the PLCT and FLOPLCT methods are shown in Fig 8B, respectively.

#### Remarks

The first time PLCT and FLOPLCT time-frequency representation of the signal *x*(*τ*) are shown in Fig 6A and 6B under Gaussian noise environment (*SNR* = 5*dB*), respectively. Fig 6C and 6D are the second time time-frequency representation of the signal *x*(*τ*) employing the PLCT and FLOPLCT methods, respectively. The third time PLCT and FLOPLCT time-frequency representation of the signal *x*(*τ*) are given in Fig 6E and 6F, respectively. The corresponding polynomial chirp rate parameters are shown in Table 2. It can be seen that the first time PLCT time-frequency distribution is actually STFT time-frequency distribution, and the first time FLOPLCT time-frequency representation is FLOSTFT time-frequency representation, where the polynomial chirp rate parameters is [0 0 0 0]. The polynomial chirp rate parameters of the second time and the third time PLCT time-frequency distribution are [30.08907 3.98387 0.33051–0.03301 ] and [30.01061 3.99532 0.33404–0.03337 ], respectively. The polynomial chirp rate parameters of the second time and the third time FLOPLCT time-frequency distribution in Table 2 are [30.08394 3.97367 0.33268–0.03311 ] and [30.00318 3.99755 0.33384–0.03336 ], respectively. The estimated polynomial parameters are very close to the actual parameter values [30 4 1/3 1/30]. Compared with the first time method (STFT and FLOSTFT), the second time and the third time PLCT and FLOPLCT time-frequency methods based on the polynomial parameters have better time-frequency resolution in Gaussian noise environment, which demonstrates the advantages of the polynomial method.

The first time, the second time and the third time PLCT and FLOPLCT time-frequency representation of the signal *x*(*τ*) are shown in Fig 7A–7F under *SαS* stable distribution noise environment (*MSNR* = 16*dB*, *α* = 0.8), respectively. The corresponding polynomial chirp rate parameters are shown in Table 3. The simulation results show that the first time PLCT (STFT method), the second time and the third time time-frequency distribution fail in *SαS* stable distribution noise environment, and their estimated polynomial parameters are [0 0 0 0], [33.34755 7.78413–0.54496 0.01095 ] and [30.44407 4.66236 0.15859–0.02324 ], respectively. It is shown that the deviation between the estimated polynomial parameters and the actual parameters is large. The three times PLOPLCT also can demonstrate time-frequency distribution of the signal *x*(*τ*) well under *SαS* stable distribution noise environment, and the second time and the third time estimated polynomial parameters [30.06923 4.00091 0.32692–0.03281 ] and [29.99945 3.99742 0.33395–0.03336 ] are very close to the actual parameter under low *MSNR*. Hence, the results show the PLCT method can only work in Gaussian noise environment, but the FLOPLCT method can work in Gaussian noise environment and *α* stable distribution noise environment, which has good toughness.

Fig 8A shows that *MSEs* of the three times polynomial parameters estimation of the improved FLOPLCT time frequency representation method are stable between -22*dB* and -30*dB* when *α* changes from 0.6 to 2, but the three times polynomial parameters estimation of the improved PLCT time frequency representation method change from 20*dB* to -30*dB*. Especially, when *α* < 1.4, the advantage of the FLOPLCT method is more obvious. Hence, the polynomial parameters estimation *MSEs* of the improved FLOPLCT method are lower than the PLCT method under *α* stable distribution noise. Fig 8B shows that *MSEs* of the three times polynomial parameters estimation of the improved FLOPLCT time frequency representation method are lower than those of the PLCT method when *MSNR* changes from 8*dB* to 22*dB*, the mixed *MSEs* of the PLCT method are large and the change is irregular, however, the mixed *MSEs* of the FLOPLCT time frequency representation method are stable between -23*dB* and -30*dB*. The above results demonstrate the advantages of the proposed FLOPLCT method.

The FLOLCT method in Section 3.1 has a constant chirp rate, so its time-frequency resolution is fixed, as shown in Figs 4 and 5. The FLOPLCT method uses a set of polynomial chirp rate *c*_1_, *c*_2_… *c*_*n*-1_, *c*_*n*_ and time-frequency iteration times to improve the resolution of time-frequency representation, as shown in Figs 6 and 7. At the same time, we can also see the chirp rate parameters of each time time-frequency distribution from Tables 2–3. By comparing the mixed *MSEs* of the polynomial chirp rate parameters of the PLCT and FLOPLCT methods in different *α* and MSNR in Fig 8, we can see the error accuracy of polynomial parameter estimation of the PLCT and FLOPLCT time-frequency distribution methods from different characteristic index *α* stable distribution and *MSNR* environment, the smaller *MSEs* is, the closer the chirp rate parameters are to the actual value, and the performance of the algorithm is better. Aimming at the calculation of the FLOPLCT time-frequency distribution, the polynomial chirp rate parameters can be set with a large order *n* at the beginning, when the time-frequency representation obtained by iteration can be well approximated by a low-order polynomial chirp rate parameters, then the later higher-order chirp rate parameters will be approximately equal to 0.

## Application simulations

**Fig 9:**
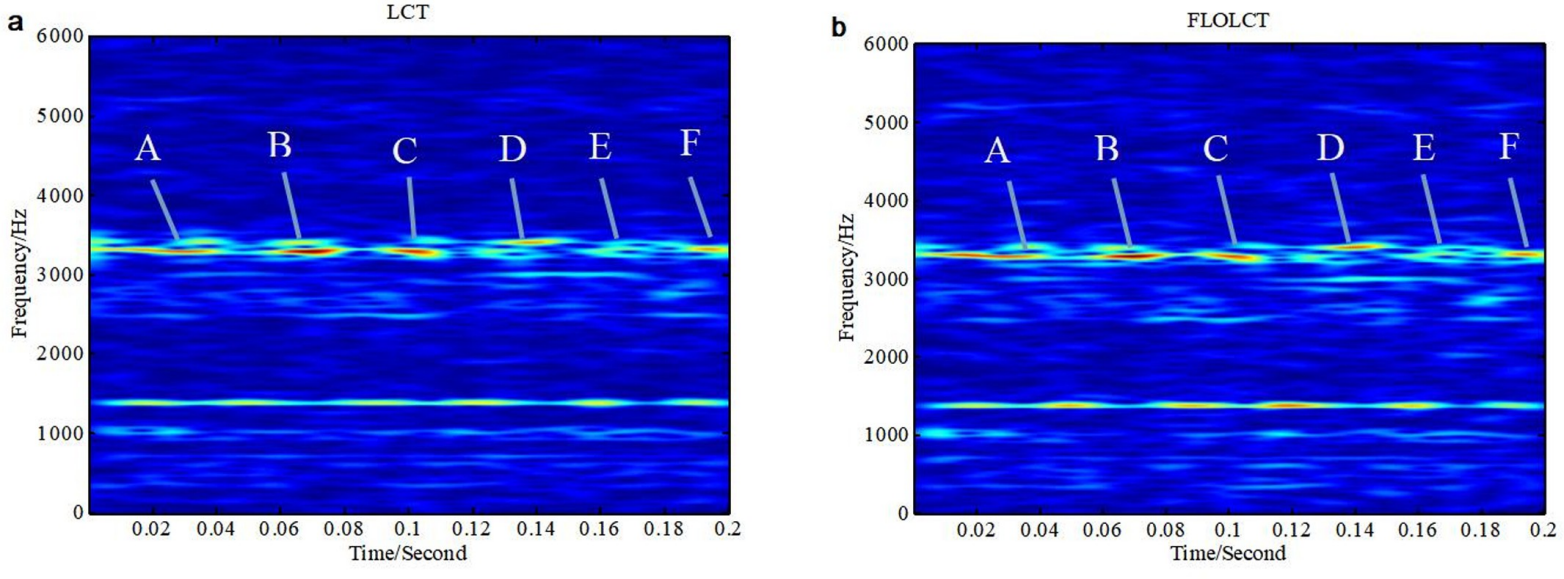
The time-frequency representations of the bearing ball fault in the position of DE data contaminated by Gaussian noise (*SNR* = 5*dB*). (a. LCT time-frequency representation (*c* = 5); b. FLOLCT time-frequency representation (*c* = 5, *p* = 1.2)).

**Fig 10:**
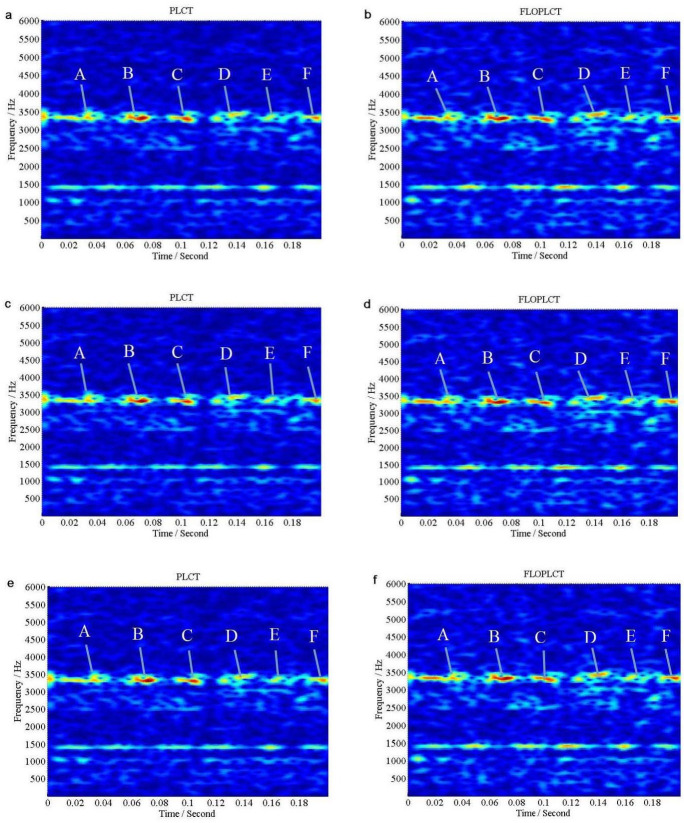
The time-frequency representations of the bearing ball fault in the position of DE data contaminated by Gaussian noise (*SNR* = 5*dB*). (a. The first time PLCT time-frequency representation; b. The first time FLOPLCT time-frequency representation; c. The second time PLCT time-frequency representation; d. The second time FLOPLCT time-frequency representation; e. The third time PLCT time-frequency representation; f. The third time FLOPLCT time-frequency representation).

**Fig 11:**
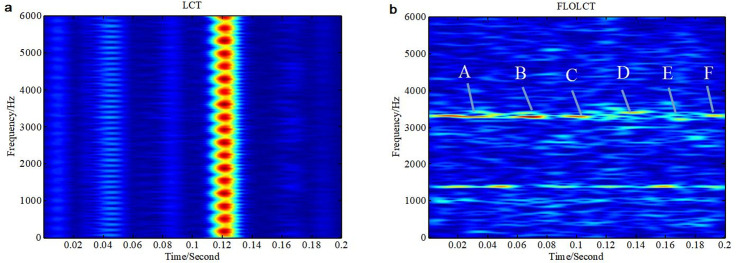
The time-frequency representations of the bearing ball fault in the position of DE data contaminated by *α* stable distribution noise (*MSNR* = 18*dB*, *α* = 0.8). (a. LCT time-frequency representation (*c* = 5); b. FLOLCT time-frequency representation (*c* = 5, *p* = 1.2)).

**Fig 12:**
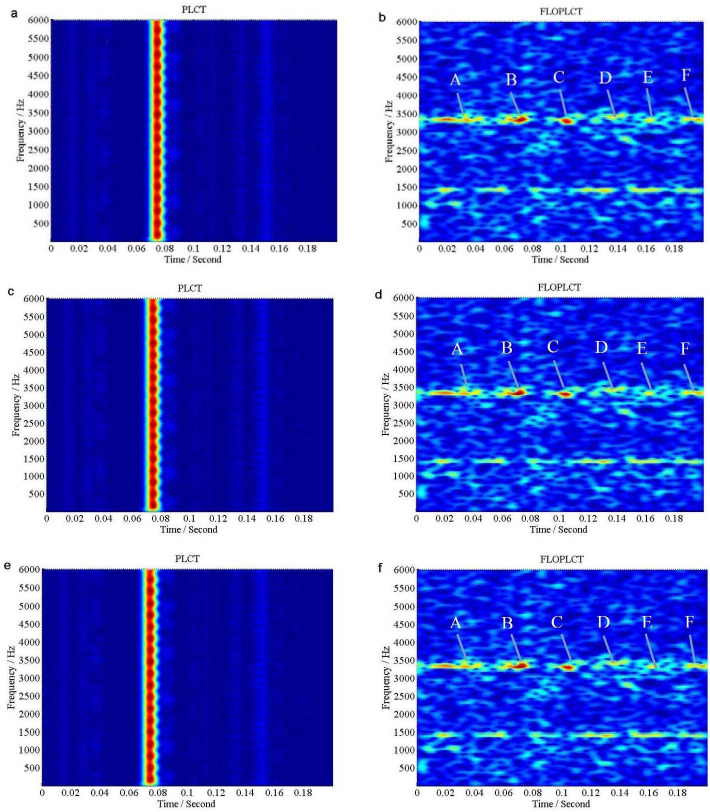
The time-frequency representations of the bearing ball fault in the position of DE data contaminated by *α* stable distribution noise (MSNR = 18*dB*). (a. The first time PLCT time-frequency representation; b. The first time FLOPLCT time-frequency representation; c. The second time PLCT time-frequency representation; d. The second time FLOPLCT time-frequency representation; e. The third time PLCT time-frequency representation; f. The third time FLOPLCT time-frequency representation).

PDFs of the inner race and outer race fault data in the vibration position of DE, FE and BA have strong impulse and serious trailing end because of the local defects of rolling element bearings, as shown in Table 1 and Fig 3D, 3H and 3J. The fault data are non-Gaussian and non-stationary *α* stable distribution because of the presence of impulses. In this section, the test data adopt from the normal DE signal (1772 rpm) and the ball DE fault (1729 rpm), and 0.2 seconds data is selected as the test signal, then *N* = 1400. The existing LCT and PLCT, the improved FLOLCT and FLOPLCT time-frequency representation have been applied to analyze the data contaminated by Gaussian or *α* stable distribution noise to demonstrate the vibration characteristics of the bearing with an artificially seeded defect on ball in the position of DE. The simulation results are shown in Figs 9–12 and Tables 4 and 5.

**Table 4:** The estimated polynomial parameters of the bearing ball fault in the position of DE data contaminated by Gaussian noise (*SNR* = 5*dB*) employing the PLCT and FLOPLCT methods (*n* = 3).

Polynomial parameters	PLCT			FLOPLCT		
Estimated *c*	n = 1	n = 2	n = 3	n = 1	n = 2	n = 3
*c*_*1*_	3478.8	3503.1	3503.1	3289.3	3231.6	3231.6
*c*_*2*_	1.7	1.8	1.8	1.4	1.2	1.2
*c*_*3*_	0	0	0	0	0	0

**Table 5:** The estimated polynomial parameters of the bearing ball fault in the position of DE data contaminated *α* stable distribution noise (*MSNR* = 16*dB*, *α* = 0.8) employing the PLCT and FLOPLCT methods (*n* = 3).

Polynomial parameters	PLCT			FLOPLCT		
Estimated *c*	n = 1	n = 2	n = 3	n = 1	n = 2	n = 3
*c*_*1*_	3897.4	3332.3	4396.5	3459.9	3505.9	3505.9
*c*_*2*_	3.8	2.2	3.1	1.7	1.9	1.9
*c*_*3*_	0	0	0	0	0	0

Fig 9A and 9B are the time-frequency representations of the bearing ball fault in the position of DE data contaminated by Gaussian noise (*SNR* = 8*dB*) employing LCT (*c* = 6) and FLOLCT (*c* = 6, *p* = 1.2, *p* = 1.2), respectively. It can be seen that the LCT and FLOLCT methods can estimate the time-frequency representations of the bearing ball fault well. The LCT and FLOLCT time-frequency representations of the bearing ball fault in the position of DE data contaminated by *α* stable distribution noise (*α* = 0.8, *MSNR* = 18*dB*) are shown in Fig 11A and 11B, respectively. The simulation results show that the LCT method is invalid, but the FLOLCT method can demonstrate the time-frequency representations of the bearing ball fault well. Hence the FLOLCT method has a wider range of applications, and which has better performance.

In Fig 10 we show from top to bottom: the first time PLCT time-frequency representations of the bearing ball fault in the position of DE data contaminated by Gaussian noise (*SNR* = 5*dB*); the first time FLOPLCT time-frequency representation of the bearing ball fault; the second time PLCT time-frequency representation of the bearing ball fault; the second time FLOPLCT time-frequency representation of the bearing ball fault; the third time PLCT time-frequency representation of the bearing ball fault; the third time FLOPLCT time-frequency representation of the bearing ball fault. The corresponding three time polynomial chirp rate parameters employing the PLCT and FLOPLCT methods are shown in Table 4. The first time PLCT time-frequency distribution is actually STFT time-frequency distribution, and the first time FLOPLCT time-frequency representation is FLOSTFT time-frequency representation, where the polynomial chirp rate parameters is [0 0 0]. The polynomial chirp rate parameters of the second time and the third time PLCT time-frequency distribution are [3478.8 1.7 0] and [3503.1 1.8 0], respectively. The polynomial chirp rate parameters of the second time and the third time FLOPLCT time-frequency distribution are [3289.3 1.4 0] and [3231.6 1.2 0], respectively. Compared with the first time method (STFT and FLOSTFT), the second and third times time-frequency representation employing the PLCT and FLOPLCT methods have better time-frequency resolution. Hence, the PLCT and FLOPLCT methods can better demonstrate the fault characteristics of the bearing ball fault data in the position of DE than the STFT and FLOSTFT methods, which reveals the advantages of the polynomial methods.

The first time, the second time and the third time PLCT and FLOPLCT time-frequency representation of the bearing ball fault in the position of DE data contaminated by *α* stable distribution noise (*MSNR* = 18*dB*) are shown in Fig 12A–12F, respectively. The corresponding polynomial chirp rate parameters are shown in Table 5. The simulation results show that the first time PLCT (STFT method), the second time and the third time time-frequency distribution are invalid because of *SαS* stable distribution noise environment, and their estimated polynomial parameters are [3897.4 3.8 0], [3332.3 2.2 0] and [4396.5 3.1 0], respectively, it is shown that the estimated polynomial parameters vary greatly. Three times PLOPLCT can demonstrate time-frequency representation of the bearing ball fault well, and three times estimated polynomial parameters [3459.9 1.7 0], [3505.9 1.9 0] and [3505.9 1.9 0] are very close, which shows the stability of the improved PLOPLCT method. Hence, the improved FLOPLCT method has better time frequency resolution for the bearing fault analysis and diagnosis, and which can be appropriate for Gaussian noise and *α* stable distribution noise environment, has good toughness.

From Figs 9, 10B, 11, 12B, 12D and 12F, we can see that the frequency component of the bearing ball fault in the position of DE data regularly changes, and which includes 1400 Hz and 3400 Hz frequency component. The time interval between the vibrations in A, B, C, D, E and F is about 30 milliseconds, then the characteristic frequency of the bearing ball fault is approximately 33Hz. Hence, the improved FLOLCT and FLOPLCT methods have better performance superiority than the existing LCT and PLCT methods, the improved methods are more suitable for the bearing fault auxiliary analysis diagnosis under complex environment, and which are robust.

In the complex situation of unknown environment, we can first use the FLOPLCT time-frequency method for the actual bearing fault diagnosis and analysis with toughness. In view of the shortcomings of FLOPLCT in time-frequency analysis of multi-component signals, we can combine the FLOPLCT method with other time-frequency analysis methods, such as FLOPWVD, FLOST, FLOARMA, etc., and make comprehensive judgment based on their respective advantages to obtain better analysis results.

## Conclusions

In this paper, *α* stable distribution is used as statistical model to simulate the real bearing fault data and background noise. The improved FLOLCT time frequency representation methods is proposed employing fractional low order statistics in order that the LCT methods can be suitable for Gaussian and *α* stable distribution noise environment. The proposed FLOLCT method can provide a well-concentrated time frequency representation and resolution for the bearing ball fault analysis and diagnosis than the FLOSTFT method in *α* stable distribution environment because of the application of chirp rate *c*, and its time frequency resolution is controlled by the chirp rate *c*, when *c* = 0, the FLOLCT degrades to FLOSTFT. FLOLCT only can demonstrate higher energy aggregation for the signal whose instantaneous frequency trajectory is linear function of time. Hence, the improved FLOPLCT is proposed employing a series of polynomial chirp rate parameters $\tilde{c}=c_{1},c_{2}\ldots c_{n-1},c_{n}$. The FLOPLCT method can give optimal polynomial chirp rate parameters according to the last time frequency peak of the signal in time frequency domain. when $\tilde{c}=c$, FLOPLCT degenerates into FLOLCT. We compare the performance of the existing LCT and PLCT methods, the improved FLOLCT and FLOPLCT methods. The results show that the performance of the FLOLCT and FLOPLCT methods are better than the LCT and PLCT methods, which can effectively suppress *α* distribution noise, and work in low *MSNR*. In practical applications, the fault signature of the bearing ball fault in the position of DE data is extracted in time frequency representation employing the FLOLCT and FLOPLCT methods, the results show their performance advantages. The FLOPLCT method is not very effective in multi-signal analysis, and the time-frequency effect is not very satisfactory for signals with large frequency variations. The edge of the signal energy concentration has certain daub, which needs to be further improved.
